# The effectiveness of a stratified group intervention using the STarTBack screening tool in patients with LBP - a non randomised controlled trial

**DOI:** 10.1186/1471-2474-14-342

**Published:** 2013-12-05

**Authors:** Susan E Murphy, Catherine Blake, Camillus K Power, Brona M Fullen

**Affiliations:** 1 School of Public Health, Physiotherapy and Population Science, University College Dublin, Dublin, Ireland; 2 BackCare Programme, Orthopaedic Department, Waterford Regional Hospital, Waterford, Ireland; 3 Pain Service, Adelaide and Meath Hospital, Tallaght, Dublin 24, Ireland

**Keywords:** Low back pain, Stratification, STarTBack tool, Group exercise/education Intervention

## Abstract

**Background:**

Low back pain (LBP) is costly to society and improving patient outcomes is a priority. Stratifying LBP patients into more homogenous groups is advocated to improve patient outcome. The STarT Back tool, a prognostic screening tool has demonstrated efficacy and greater cost effectiveness in physiotherapy settings. The management of LBP patients in groups is common but to date the utility of the STarT Back tool in group settings has not been explored. The aim of this study is to determine if the implementation of ‘stratified care’ when delivered in a group setting will lead to significantly better physical and psychological outcomes and greater cost effectiveness in LBP patients compared to a bestcare historical control group.

**Methods/Design:**

This study is a non randomised controlled trial. Low back pain patients recruited from the Waterford Primary Care area (population = 47,000) will be stratified into low, medium or high risk of persisting symptoms using the STarT Back Tool. Low risk patients will be offered a single one off education/exercise class offering positive messages on LBP management in line with recommended guidelines. Medium risk patients will be offered a 12 week group exercise/education intervention addressing their dominant physical obstacles to recovery. A 12 week group cognitive behavioural approach will be delivered to the high risk patients, characterised by the presence of high levels of psychosocial prognostic factors. These patients will be compared with a historical control group where therapists were blinded as to the risk stratification of patients and a generic group intervention was delivered to all patients, irrespective of their initial risk stratification. The primary outcome measure will be disability (Roland Morris Disability Questionnaire). Secondary outcomes will include back pain intensity (Visual Analogue Scale), distress (Distress and Risk Assessment Method), back beliefs (Back Beliefs Questionnaire), health status (Euroqol), global benefit (7 point likert scale), satisfaction (7 point likert scale), cost effectiveness and functional status. Outcome will be measured at baseline, 12 weeks and 6 months.

**Discussion:**

This paper details the rationale, design, methods, planned analysis and operational aspects of a study examining the utility of the STarT Back Tool as a ‘stratification tool for targeted treatment’ in a group intervention.

**Trial registration:**

Current controlled trials: ACTRN12613000431729.

## Background

Low back pain (LBP) is a major public health problem, costly to society and can lead to disabling symptoms for a large percentage of people. Symptoms are persistent, and though many LBP patients cease consulting their General Practitioner (GP) within a relatively short timeframe, 60-80% of people still report pain and disability one year later [1]. With regards to the management of LBP, guidelines recommend that patients are initially offered conservative management in the form of education and exercise/manual therapy/acupuncture or a combination of a physical and psychological treatment if individual interventions prove ineffective [2]. Though the efficacy of these treatment interventions has been established, in general outcomes from studies are at best moderate [3,4], and improving patient outcomes has become a priority amongst researchers.

Traditionally LBP has been broadly categorised into three main groupings: non specific LBP (94%), nerve root pain (5%) and red flag pathology (1%) [5]. The majority of patients with LBP are classified as ‘non specific’ and a generic treatment pathway is administered to all, irrespective of the underlying condition or psychosocial status. It has been argued that this broad classification of LBP is too heterogeneous and that patients with non specific LBP should be sub-divided into more homogenous treatment groups [6,7]. Sub-grouping and delivering targeted treatment interventions has become a key focus for researchers [8-10]. A number of classification systems have been proposed including diagnostic, prognostic and treatment based systems [11,12]. It has been well established that psychosocial factors can lead to the persistence of LBP: low mood, anxiety, catastrophisation and fear avoidance [13,14]. Bearing this in mind, Hill et al. [15] developed a prognostic screening tool the ‘STarT Back’ Tool which stratifies patients on the presence of potentially modifiable physical and psychological prognostic indicators for persisting disabling low back symptoms. Patients are stratified as low, medium or high risk of persisting symptoms and treatment targeted accordingly. The STarT Back Tool was developed in the primary care setting. It is simple to administer, patient friendly, and provides a welcome alternative to the more in-depth psychosocial questionnaires which can be cumbersome in the primary care setting [16]. The efficacy of the STarT Back screening tool has been demonstrated in individual patient management in the physiotherapy setting with both greater clinical and cost effectiveness reported in the targeted subgroups compared to usual care [10]. In view of its proven utility, the STarT Back Tool is gaining more widespread use [17-19] and its inclusion in spinal care pathways has been recommended by the recently formed UK National Spinal Taskforce [20].

Within physiotherapy settings, different models of care exist for the delivery of LBP interventions. The use of group interventions incorporating rehabilitation and promoting self management strategies are well established [2,4]. The reported benefits of group intervention over individual treatment include greater throughput of patients, treatment is more standardised which enhances outcome measurement, a self management approach to treatment is fostered and the delivery of group interventions has proved more cost effective [21]. From a patient’s perspective, it proves enjoyable and allows patients to meet others with a similar disposition [22,23]. To date, the STarT Back tool has demonstrated efficacy when physiotherapy treatment has been administered on an individual basis. This study aims to explore the utility of the STarT Back Tool in a group setting.

## Methods/Design

### Aims

The main objective of this study is to determine if the implementation of ‘stratified care’ when delivered in a group setting will lead to significantly better physical and psychological outcomes in a group of patients with non specific LBP, compared to a matched best care historical control. The historical control group will consist of individuals who attended the Back Pain Clinic between 2008 and 2011. Evaluation of treatment effect will be made within each of the stratified treatment groups and comparisons will be made between the stratified group outcomes and best care historical control. Secondary analysis will compare the delivery costs of the historical and stratified interventions.

### Hypotheses

The following hypotheses will be tested.

1 Patient stratified to the ‘high risk’ intervention based on the STarT Back tool will have better physical and psychological outcomes compared with their baseline scores and compared with the high risk historical control group.

2 Patients stratified to the ‘medium’ and ‘low risk’ groups based on the STarT Back tool will have significantly better physical and psychological outcomes compared with their baseline scores and equally good outcomes compared with the best-care historical control.

### Study design

This study is a non randomised controlled trial comparing a new stratified intervention to a historical control treatment, which is usual non-stratified care. The trial is thus pragmatic in nature and aims to establish the effectiveness of a treatment intervention in routine everyday practice [24]. The study will determine if the implementation of stratified care in a group setting, using the STarT Back risk stratification tool will result in significantly better physical and psychological outcomes in LBP patients, compared with historical control data collected between 2009–2011 [25]. Patient assessments will occur at baseline, at 12 weeks and 6 months following commencement of the treatment intervention. The primary endpoint for analysis is 6 months. Recruitment will take place between February 2012 and June 2013.

This type of study design may be at risk of selection bias, but the choice of historical controls was made to allow comparability of interventions within a single clinical centre, thus removing the risk of differences in management between centres. Furthermore, since the new treatment paradigm necessitated a shift in management approach to include cognitive behavioural principles with subsequent staff training, the selection of historical controls averted any possible loss of fidelity of the routine care treatment which might have occurred if controls were recruited concurrently to the new intervention group. Other measures taken to minimise potential differences between the intervention and control treatments include; the control and historical interventions will be delivered to the same local population, referred from the same sources, using standard diagnostic and referral criteria. Both the historical and new interventions will be applied in the same clinical setting by therapists who will apply the pre and post treatment assessments in a standardised manner. The same end points will be chosen for the stratified treatment group as has been the practice for the historical controls and there will be no time lag between the historical data collection and the stratified intervention phase. This can be considered an appropriate design in the current study setting, where we have a large dataset documenting existing routine evaluation of clinical treatment outcomes [25]. Here the comparison of interest is between the existing standard management of LBP (the historical control group), in a single centre, to a new paradigm of care (intervention).

### Patient recruitment, consent, allocation

**Figure 1:**
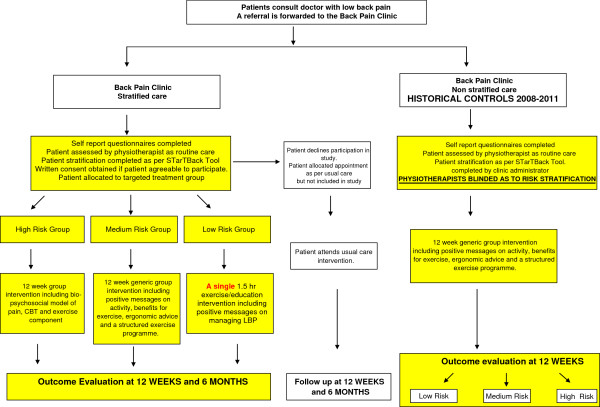
Study flow chart.

Patients will be recruited from 10 GP practices and their associated physiotherapy services within the Waterford City Primary Care area. The Waterford City Primary Care area serves a population of approx 47,000 people [26]. Potential patients will be identified (based on the inclusion criteria) when they consult their GP practice or primary care physiotherapy team and subsequently will be referred to the Back Pain Clinic at Waterford Regional Hospital. On receipt of the referral, the Back Pain clinic will forward an appointment to the patient, in addition to a copy of the self report baseline questionnaires as per usual care and written information regarding the study. On the initial screening day, the ‘STarT Back’ screening tool will be administered and patients stratified according to their level of risk of persisting symptoms - low, medium or high risk. Patients will be assessed as routine care. Routine care will include history taking, a physical examination, review of completed self report questionnaires and performance of timed functional outcome measures. Following the clinical assessment, if patients are deemed suitable for the study, a full explanation will be given by the principle investigator and any patient concerns addressed. If the patient is then willing to participate, written consent will be obtained (Figure 1).

The historical control consists of patients who attended the Back Pain Clinic between 2009–2011. These patients were referred from the 10 GP practices and their associated physiotherapy services within the Waterford City Primary Care area in exactly the same manner as the intervention group. The patients completed a copy of the same self report questionnaires as the intervention group. In the control group, risk stratification was performed by an independent party (administrator at the clinic) after the initial assessment using the STarT Back scoring algorithm. The therapists were blinded as to the risk stratification of each patient until completion of the treatment intervention at three months. Patients in the historical control group were assessed as routine care which included history taking, a physical examination, review of completed self report questionnaires and performance of timed functional outcome measures in exactly the same manner as the intervention group. Following initial assessment if patients were deemed suitable, all patients irrespective of their risk stratification were entered into a generic 12 week group exercise/education intervention. Consent was obtained retrospectively for the inclusion of these historical controls in line with the National Consent Policy [27].

### Eligibility criteria

Patients (male and female) with chronic non specific LBP of more than three months duration and aged 18–65 years will be included. Full details of inclusion/exclusion criteria are summarised in Table 1.

**Table 1:** Study inclusion and exclusion criteria

**Criteria**	
**Inclusion**	
Age	18–65 years
Diagnosis	Mechanical low back pain
Duration	Greater than 3 months duration
Language	English speaking and English literate
Consent	Willing and able to give full consent
**Exclusion**	
Pathology	Suspected or confirmed serious spinal pathology (cauda equine syndrome, fracture, metastatic, inflammatory spinal disease, pre-existing neurological condition)
Past medical history	Illicit drug use, spinal surgery, spinal cord stimulator implantation, cardiac history, psychiatric illness or personality disorder, intellectual disorder
Pregnancy	Suspected or confirmed pregnancy

### Ethical approval

Ethical approval was obtained from Waterford Regional Hospital Ethics committee Health Service Executive (HSE) South in June 2011.

### Screening tool

The STarT Back Prognostic screening Tool allows clinicians to stratify patients based on the presence of potentially modifiable physical and psychological prognostic indicators for persistent disabling symptoms [28]. Patients are stratified into one of three categories: ‘low risk’, ‘medium risk’ or ‘high risk’ of persisting symptoms. The tool consists of 9 items and includes constructs on referred pain, co-morbid pain, disability (2 items), bothersomeness, catastrophising, fear, anxiety and depression. Questions 5–9 encapsulate psychosocial features associated with persistent LBP (psychosocial subscale). The maximum score is 9. Patients are asked to agree or disagree with statements numbered 1–8 and a score of 1 is allocated to each statement the patient agrees with. The 9th item relates to bothersomeness and a score of 1 is allocated if the patient scores either ‘very much’ or ‘extremely’. Patients are classified as low risk of persisting symptoms if they score 0–3 and high risk if scoring 4 or 5 on the psychosocial subscale. All intermediate patients are classified as medium risk. High risk patients are characterised by the presence of high levels of psychosocial prognostic factors, with or without the presence of physical factors whereas medium risk patients are characterised by the presence of physical and some psychosocial prognostic factors, but the physical features are more dominant. Treatment intervention is directed dependent on the baseline risk stratification [10]. The tool has been validated against well established questionnaires regarding disability and psychological parameters and it psychometric performance was shown to be similar to that of the longer Orebro Musculoskeletal Screening Questionnaire [15,29].

### Treatment interventions

Following assessment patients will be streamed into one of three treatment interventions based on the STarT Back tool. All interventions will be grouped based and delivered by physiotherapists employed in the Back Screening Clinic.

#### Low risk intervention

Patients will attend a single 1.5 hour small group session delivered by the physiotherapist promoting active management of their back symptoms and outlining positive messages on maintaining a healthy spine in line with the ‘The Back Book’ [30]. This session will be conducted within a biopsychosocial framework and it’s aim will be to normalise the episode of LBP in line with the guidelines [2]. The benefits of exercise will be emphasised, advice to increase activity levels will be given and information on local exercise centres will be provided. Patients will be instructed with an exercise regime and given a manual which contains information on LBP management, the back exercises and a home exercise log. Each group will consist of three to five patients. The class will also offer the opportunity for patients to ask questions regarding their LBP and self-management. Patients will be encouraged to become more active in their lifestyles and to perform the back exercises on a regular basis.

#### Medium risk intervention

Patients in this group present with predominantly physical prognostic indicators (disabling LBP, referred leg pain and co-morbid pain) without high levels of psychosocial distress. Patients will attend four 90-minute group exercise/education sessions over four weeks. The content of the educational sessions will include positive messages on managing LBP based on ‘The Back Book’ [30], discussion on the benefits of exercise, a review of spinal anatomy, direction on lifting and handling, ergonomic advice, weight management and the impact of stress, anxiety and low mood on LBP. These sessions will be conducted with a biopsychosocial framework. Patients will be instructed with a stability exercise programme. Each class is a progression from the previous one. All patients will receive a ‘programme’ manual containing general educational information, instruction in each exercise and a home exercise log. Patients will be encouraged to perform the exercises independently at home at least four times/week and to complete the exercise log. Patients will also be encouraged to become more active in their lifestyle and activities such as walking, cycling, swimming or other forms of cardiovascular exercises are encouraged and logged in the activity diaries. Each group will consist of six to ten patients. Patients will maintain the exercise regime independently at home for a further eight weeks following the classes and will subsequently be reviewed at the clinic at 12 weeks. Adherence to the progamme will be monitored by reviewing the patients exercise log weekly and class attendance.

#### High risk intervention

Patients in this group display high levels of psychosocial distress around their LBP including elements of anxiety, low mood or fear. Physiotherapists administering this component of the intervention will receive extra training in cognitive behavioural therapy (CBT). Patients will attend four 120 min group sessions. The content of this intervention is modelled on the ‘high risk’ intervention delivered in the original STarT Back Trial [10]. Sessions will utilise cognitive behavioural strategies to address unhelpful beliefs and behaviours around LBP. The CBT intervention will include discussion of the bio-psychosocial model of pain, the impact of persistent pain, goal setting based around patient’s functional and social limitations, graded activity, relaxation training, relapse management. It will give the patient’s a better understanding of how they can help themselves and will focus particularly on behaviour change. It will teach the patients to identify and change maladaptive behaviours and use cognitive techniques to identify and challenge unhelpful and unrealistic thoughts and beliefs. This will promote positive behaviour and beliefs about physical activity and avoidance behaviour. Specific psychological and practical skills to promote self management of pain will be developed. Each session will also include an exercise component similar to the medium risk intervention and each patient will receive a ‘programme’ manual containing the relevant information and exercises. Patients will be encouraged to perform the exercises independently at home at least four times per week as well as adopting CBT principles e.g. pacing and goal setting. Each group will consist of four to six patients. Patients will continue the exercise regime independently at home for 8 weeks following the classes and will be reviewed at the clinic at 12 weeks. Adherence to the programme will be monitored by reviewing the patients exercise log weekly and class attendance.

#### Best care historical control

The historical control consists of patients who attended the clinic between 2009–2011 [25]. Patients were stratified using the STarT Back screening tool but a non-targeted (‘one treatment fits all’) model of care was delivered to patients. All patients undertook the same generic 12 week group education/exercise programme promoting self management and rehabilitation. The efficacy of this intervention has been established [25] and subsequently this template was utilised to develop the medium and high risk interventions. Similar to the medium and high risk interventions, the historical control comprised two phases. In phase one, patients attended once weekly for four weeks and each class was 1.5 hours in duration. Sessions included exercise and back educational information similar to the ‘medium risk’ intervention already described. In phase two, patients continued independently with the structured home exercise programme for a further eight weeks. Each group consisted of six to ten patients. Validated self report and objective physical outcome measures monitored progress at baseline and at 12 weeks. Outcomes utilised included disability (Roland Morris Disability questionnaire (RMDQ)) [31], pain (Visual Analogue Scale (VAS)) [32], distress (Distress and Risk Assessment Method (DRAM)) [33], back beliefs (Back Beliefs Questionnaire (BBQ)) [34], Simmond’s Functional Tests [35] and lumbar flexibility [36].

### Study assessment tools

#### Baseline assessment

A schematic view of the outcome measures and assessment time lines is presented in Table 2.

**Table 2:** Patient outcome measures and timelines

	**Domain**	**Outcome measures**	**Time-points**
			**(months)**
	Demographics	Age, gender, duration of LBP, employment status, educational attainment, medico-legal status	0
Primary	Back pain disability	Roland and Morris Disability Questionnaire	0,3,6
Secondary	Pain	Visual analogue scale	0,3,6
	Distress	Distress and Risk Assessment Method	0,3,6
	Beliefs	Back Beliefs Questionnaire	0,3,6
	Preference based health utility	EQ-5D	0,3,6
	Global perceived benefit	Compared to symptoms at baseline (7 point likert scale)	0,3,6
	Satisfaction scale	Satisfaction with care received (7 point likert scale)	0,3,6
	Objective spinal mobility	Range of motion T-12 & S1	0,3
	Objective physical function	Simmond’s Tests:	0,3
		sit-stand, forward reach, 50-foot walk test	

#### Follow up assessment

Patients will be re-assessed at the clinic at week 12 for follow up of primary and secondary outcome measures. At 6 months, patients will be followed up by postal questionnaire. Maximising completion of follow-up questionnaires will be achieved by (1) texting the patient in advance to inform them that the questionnaire is being sent, (2) enclosing a pre-paid self addressed return envelope, (3) following up with the patient by phone once they have received the questionnaire.

#### Outcome measures

The outcome measures assess the domains of disability, pain, psychosocial and health status, global perceived benefit, satisfaction and functional status (Table 2). The primary outcome measure will evaluate change in functional disability due to LBP measured by the Roland Morris Disability Questionnaire (RMDQ). The RMDQ is widely used in LBP studies in Primary Care and has demonstrated good validity, reliability and responsiveness [31]. All outcome measures demonstrate proven validity and reliability in their particular domain - pain (VAS 0–10) [32], anxiety and depression (DRAM) [33], back beliefs (BBQ) [34], global perceived benefit scale and satisfaction scale (seven point likert scale) [37]. Functional outcomes include sit to stand test, functional reach test and 50 ft. walk test [35] and lumbar spine flexion range of motion [36].

With respect to cost analysis, each patient will be asked to complete a cost diary documenting their medication costs and health care costs (direct costs) for a retrospective three months, prior to attending the clinic and for the prospective six months while participating in the study. The health economic evaluation will consist of a cost benefit analysis and a cost utility analysis, with utility measured by the EuroQol (EQ-5D) [38]. The cost of each treatment strategy will be determined prospectively and includes staff time, overheads and equipment.

### Analyses

All data will be entered into the IBM Statistical Package for the Social Sciences [39] and subsequently cleaned and checked for normality. Baseline differences between the treatment groups will be explored with independent t tests and chi square tests. Preliminary analysis will compare pre to post intervention outcomes for each treatment group to determine if a significant treatment effect is achieved by each of the stratified interventions using paired t-tests and Cohen’s d effect size statistics. The main analysis will compare treatment response between the stratified and control groups using analysis of covariance (ANCOVA) models with baseline measures as the covariate and treatment group as the between group factor. Statistical significance will be set at p < 0.05. Missing data will be imputed using the linear and logistic regression models applied by the multiple imputation functions in SPSS and an intention to treat analysis will be performed. We will also conduct a complete case analysis for those cases where we have complete follow up data. In the event of non normal data, non parametric tests will be performed instead. Exploratory economic analyses will be undertaken. Costs of each intervention will be calculated and quality-adjusted life years (QALYs) gained computed. These will be compared between interventions using independent t tests. Six month health care utilisation attendances and associated costs will be calculated for each patient and compared between the intervention groups using chi square tests for categorical data and t tests for continuous data.

### Sample size and statistical power

The RMDQ has been chosen as the primary outcome measure in keeping with prior research. Although a change of 2.5 points on the RMDQ is considered to be a minimum clinically important difference [40], prior research has based sample calculations on a between group difference of 1.8 points [4]. As the high risk intervention is the new intervention, our first hypothesis will test the superiority of ‘stratified treatment’ over previous best care for the high risk subgroup. Following statistical review of both the original STarT Back trial [10] and existing data held by the researcher (RMDQ Sd_change_ = 4.7; correlation between pre and post RMDQ ρ = 0.592), sample size calculation was performed to detect an effect size of 0.38 for the new high risk intervention over controls, with 80% power and alpha (two-tailed) = 0.05. After adjusting for ANCOVA [41], the resulting minimum sample required for each of the historical and new high risk groups is 72 patients (n = 144).

Equivalence of the stratified treatment model to standard care for medium and low risk groups will be determined if the lower boundary of the 95% CI around the mean difference RMDQ does not exceed the 1.8 threshold. For this one sided 2.5% non-inferiority hypothesis, a minimum of 57 participants in each of the medium and low risk historical and new treatment groups is necessary (n = 228). Thus, the total minimum sample is 372 patients.

## Discussion

We have presented the rational and design of a study exploring the delivery of a group intervention to LBP patients where stratified care using the STarT Back psychosocial screening tool was utilised and targeted treatment implemented. Although it is generally accepted that randomised controlled trials provide the highest level of evidence, a non randomised controlled design was implemented in this study as it mirrors real life clinical practice and is the most cost neutral design for the limited resources available [24]. There are naturally limitations to non randomised controlled trials including the inability to infer that the change in outcome is directly related to the intervention. However as outlined earlier, every effort has been made to standardise the delivery of care and thereby minimise bias. The results of the study will be published once the study is concluded.

## Conclusions

This study will investigate the clinical and cost effectiveness of a group intervention for LBP patients, where patients will be ‘stratified’ using the STarT Back prognostic screening tool and a ‘targeted treatment’ approach will be delivered. The study will recruit patients from the Waterford City primary Care Area and patients will be followed up over a 6 month timeframe. Study recruitment commenced in February 2012 and is currently on target to close in June 2013. Follow up is targeted for completion in December 2013 and results will be finalised for publication by April 2014.

## Abbreviations

LBP: Low back pain; UK: United Kingdom; CBT: Cognitive behavioural therapy; RMDQ: Roland and Morris disability questionnaire; VAS: Visual analogue scale; DRAM: Distress and risk assessment method; BBQ: Back beliefs questionnaire; QALYs: Quality-adjusted life years.

## Competing interests

This study received an unrestricted educational grant from Pfizer Healthcare, Ireland, however the authors declare that they have no competing interests.

## Authors’ contributions

SM is the principal investigator. SM together with her supervisory team of CB, CKP and BMF designed the study and were responsible for the protocol. CB is responsible for the sample size and power calculation and for the design of the statistical analysis. SM will manage the project in the clinical setting. All authors read and approved the final manuscript.

## Pre-publication history

The pre-publication history for this paper can be accessed here:

http://www.biomedcentral.com/1471-2474/14/342/prepub
